# Changes in Body Composition and Strength after 12 Weeks of High-Intensity Functional Training with Two Different Loads in Physically Active Men and Women: A Randomized Controlled Study

**DOI:** 10.3390/sports10010007

**Published:** 2022-01-04

**Authors:** Daniel P. Kapsis, Athanasios Tsoukos, Maria P. Psarraki, Helen T. Douda, Ilias Smilios, Gregory C. Bogdanis

**Affiliations:** 1 School of Physical Education and Sports Science, National and Kapodistrian University of Athens, 17237 Athens, Greece; kapsis.daniel@gmail.com (D.P.K.); atsoukos@phed.uoa.gr (A.T.); 2 Department of Physical Education & Sports Science, Democritus University of Thrace, 69100 Komotini, Greece; akiro_on@hotmail.com (M.P.P.); edouda@phyed.duth.gr (H.T.D.); ismilios@phyed.duth.gr (I.S.)

**Keywords:** CrossFit, high-intensity, muscle hypertrophy, Fat Mass Loss

## Abstract

This study examined the effects of two different resistance loads during high-intensity Functional Training (HIFT) on body composition and maximal strength. Thirty-one healthy young individuals were randomly assigned into three groups: moderate load (ML: 70% 1-RM), low load-(LL: 30% 1-RM), and control (CON). Each experimental group performed HIFT three times per week for 12 weeks with a similar total volume load. Body fat decreased equally in both experimental groups after 6 weeks of training (*p* < 0.001), but at the end of training it further decreased only in LL compared to ML (−3.19 ± 1.59 vs. −1.64 ± 1.44 kg, *p* < 0.001), with no change in CON (0.29 ± 1.08 kg, *p* = 0.998). Lean body mass (LBM) increased after 6 weeks of training (*p* = 0.019) in ML only, while after 12 weeks a similar increase was observed in LL and ML (1.11 ± 0.65 vs. ML: 1.25 ± 1.59 kg, *p* = 0.034 and 0.013, respectively), with no change in CON (0.34 ± 0.67 kg, *p* = 0.991). Maximal strength increased similarly in four out of five exercises for both experimental groups by between 9.5% and 16.9% (*p* < 0.01) at the end of training, with no change in CON (−0.6 to 4.9%, *p* > 0.465). In conclusion, twelve weeks of HIFT training with either low or moderate resistance and equal volume load resulted in an equal increase in LBM and maximal strength, but different fat loss.

## 1. Introduction

Regular resistance training (RT) induces significant increases in muscle mass, strength and power [1] which, in turn, improve athletic performance and health [2]. For example, increases in muscle strength following RT are correlated with improvements in sprint [3,4], countermovement jump [5] and change in direction performance [6]. Furthermore, an increase in muscle mass due to RT also results in health-related benefits, such as enhanced energy metabolism, basal metabolic rate, and glucose tolerance [2,7,8,9,10,11]. Due to the time commitment required to perform traditional RT programs in the gym, alternative methods of training have evolved to maximize adaptations with the shortest possible duration. high-intensity interval training (HIIT) is a contemporary training method consisting of relatively short bouts of vigorous activity, separated by recovery intervals of complete rest or low-intensity exercise [12]. HIIT mainly involves running and cycling exercise lasting 20–30 min, and induces rapid metabolic and cardiorespiratory adaptations [13]. Lately, a training modality named as high-intensity functional training (HIFT) is increasingly used in gym settings. HIFT emphasizes functional multi-joint muscle-strengthening exercises (e.g., overhead squats, deadlifts, etc.), performed in a circuit fashion for a certain number of seconds or repetitions and with very short or no recovery intervals between sets [14,15,16,17]. HIFT can be tailored for various fitness levels, and has been documented to elicit greater muscle recruitment than repeated bouts of aerobic exercises, thus improving body composition, cardiovascular endurance, strength, and flexibility [15,16,17,18,19,20].

Recent studies have shown that HIFT-based programs result in significant improvements in maximal oxygen consumption ~12% [15,18], reduction in adipose tissue [15,19] and increases in bone mineral density [20]. However, to our knowledge, little is known regarding the effects of HIFT on lean body mass (LBM) and maximal strength. A recent systematic review and meta-analysis aiming to identify the effect of HIFT on body composition [14] quoted only a few studies that carried out such measurements and reported conflicting results [15,17,19,21]. For example, Feito et al. [17,21] demonstrated no statistically significant increase in LBM after 16 weeks of HIFT performed at least twice weekly. On the other hand, Murawska-Cialowicz et al. [19] demonstrated significant increases in LBM after three months of HIFT performed twice per week. One possible reason for these discrepancies may be the relative load used in HIFT programs. Notably, even in traditional RT programs, it is still debatable which training routines may promote greater hypertrophy, with numerous conflicting research data [22,23,24].

A large number of studies suggest that the most efficient load that maximizes muscle hypertrophy is between 60% and 80% of one repetition maximum (1-RM) performed in multiple sets of 8–12 repetitions [25,26,27,28]. However, recently it has been suggested that muscle hypertrophy can be attained using either moderate to high (70–80% 1-RM) or low loads (30–50% 1-RM), if repetitions are performed to exhaustion [24,29]. This versatility in load choice for muscle hypertrophy may be used to attain gains in muscle mass, without excessively loading the musculoskeletal system [24,30]. Taking into consideration the limited and conflicting results regarding changes in LBM following HIFT, and the intriguing question of whether moderate and low loads used in HIFT may be equally effective on modifying body composition, the aim of the present study was to evaluate the effect of a 12-week HIFT program using equated volumes of either low load (LL-30% 1-RM) or moderate load (ML-70% 1-RM) exercises on maximal muscle strength and body composition. Based on the recent evidence [24], we hypothesized that both loads would lead to similar increases in LBM, while maximal strength would be increased more when training with the heavier load.

## 2. Materials and Methods

### 2.1. Experimental Design

A randomized controlled design was used to examine the effects of HIFT with different loads (moderate or low) on body composition and maximal strength. Participants were randomly assigned to a group that either performed moderate-load (ML-70% 1-RM) or low-load resistance training (LL-30% 1-RM) or continued their recreationally active lifestyle and served as a control group (CON). The training program included five functional exercises performed in a circuit fashion for 30 s each, with 30 s of rest in between, totaling four rounds of five exercises per session. Volume load (load x sets x number of repetitions) was equal in the two groups, as participants performed as many repetitions as possible during the 30 s, i.e., 24–28 repetitions for the LL (30% 1-RM) and 8–12 repetitions for the ML (70% 1-RM). The training intervention lasted 12 weeks, with participants performing 3 full body workouts per week, and measurements were taken before and after 6 and 12 weeks of training. The dependent variables were body composition parameters (total body fat, bone mass and LBM) measured using bio-impedance analysis (BIA), and 1-RM strength in all exercises.

### 2.2. Participants

A total sample size of 27 participants would be necessary to detect significant differences with an effect size (ES) of 0.3. This was indicated by power analysis conducted by setting the alpha level to 0.05, the required power to 0.80 and the correlation coefficient between repeated measures was set to 0.5 (G-Power software, v.3.1.9.2).

Participants were recruited via electronic advertisements and word of mouth. All participants completed a medical history questionnaire. Inclusion criteria were (a) healthy males and females aged 20–40 years, (b) body mass index (BMI) less than 30 kg/m^2^, and (c) involved in regular recreational exercise (2–3 times per week) for at least the last 2 years prior to the study, mostly team sports and recreational bodyweight workouts. The exclusion criteria were as follows: (a) use of tobacco in the past 6 months, (b) a body mass increase greater than 2 kg during the 6 months preceding the study, (c) participation in a weight loss program over the 6 months preceding the study, (d) involvement in heavy resistance exercise aiming to increase muscle mass and maximal strength during the 6 months preceding the study, (e) use of dietary supplements and medications during the 6 months preceding the study, (f) history of endocrine or metabolic disorders as well as chronic diseases, (g) family history of early cardiac mortality.

**Figure 1:**
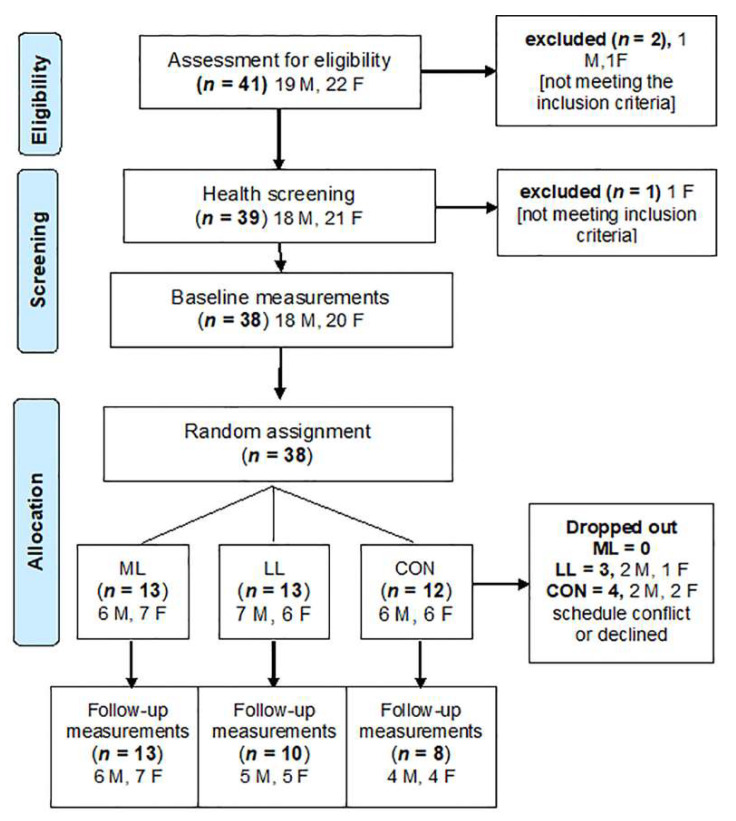
Flow diagram of the study design.

Forty-one healthy, young, physically active individuals (19 males and 22 females) took part in this study. Participants were randomly divided into three groups: two experimental (ML, LL) and one control (CON) (see Figure 1). Two of the participants were excluded during the initial screening, while eight of them did not perform >80% of the sessions or did not take part in the final measurements, as shown in Figure 1. The remaining thirty-one participants (*n* = 31) completed the study (Figure 1). All participants were fully informed about the procedures of the study, were thoroughly assessed on their medical history (chronic diseases, recent injuries, or surgery) and gave written informed consent. Ethical approval was obtained by the Institutional Ethics Committee (B1226/16-1-2020) and all procedures were in accordance with the Code of Ethics of the World Medical Association (Helsinki declaration of 1964, as revised in 2013).

### 2.3. Procedures

Training for the ML and LL groups included 3 sessions per week (Monday, Wednesday, and Saturday). The HIFT protocol consisted of four rounds of five exercises performed for 30 s each, with 30 s of recovery. Participants were encouraged to complete the maximum number of repetitions during the 30 s of exercise. In the 30 s of recovery, participants moved on to the next exercise. A 2.5-min recovery time was allowed after each round. Total session time was 25.5 min (4.5 min per round and 2.5 min of rest after each round). All aspects of the training were kept the same for both intervention groups, except the load used for each exercise. The LL group performed all exercises against a resistance of 30% 1-RM for 30 s, leading to 24–28 repetitions in each exercise. The ML group performed all exercises against 70% 1-RM, so that 8–12 repetitions were completed in 30 s. In the ten days prior to the study, both groups undertook three separate familiarization sessions, with one day of rest given in between each. During these sessions participants performed HIFT with increasing loads (from easy to moderate), which served as a preconditioning period. Then four days of rest was allowed before initiating the intervention. All training sessions of the 12-week intervention were supervised by the same two certified S&C trainers ensuring proper performance.

The following five exercises were chosen due to their common inclusion in strength training programs, and were performed in the following order: bench press, back squat, bent-over row, deadlift, and dumbbell shoulder military press. This order was chosen so as to alternate upper and lower body exercises [31].

### 2.4. Muscle Strength Assessment

The bench press exercise was performed in a supine position using an Olympic barbell (Eleiko, Halmstad, and Sweden) with a closed, pronated grip slightly wider than shoulder-width. A full repetition was counted starting with the bar over the chest with the elbows fully extended, then lowering the bar to touch the chest at approximately nipple level and finally pushing the bar upward until the elbows were fully extended [28].

The back squat was performed with the feet parallel to each other, and the Olympic barbell in a balanced position on the upper back and shoulders. The downward movement phase was performed until the thighs were parallel to the floor [28].

During the deadlift exercise, the participants placed the feet between hip and shoulder-width apart with the toes pointed slightly outward. Participants lifted the Olympic barbell off the floor by extending the hips and knees while maintaining a neutral spine position, and continued to extend the hips and knees until the body reached a fully erect torso position. A repetition was counted when returning the bar to the floor, allowing the hips and knees to flex [28].

The bent-over row exercise was performed with the feet in a shoulder-width stance, knees slightly flexed and the torso slightly above parallel to the floor. A full repetition started with the barbell hanging with the elbows fully extended, then the barbell was pulled toward the torso/lower chest, followed by full extension of the elbows [28].

The military dumbbell shoulder press exercise was performed in an upright position with two dumbbells, using a closed, pronated grip. The athletes were instructed to press the dumbbells simultaneously over the head until the elbows were fully extended, and a full repetition was counted when lowering the dumbbells to touch the clavicles and anterior deltoids and then pushing back upward until the elbows were fully extended [28].

For the assessment of 1 repetition maximum (1-RM), participants attended the laboratory having refrained from any form of exercise for more than 48 h. 1-RM was measured at baseline testing and after 6 and 12 weeks of training. A standardized procedure was followed on all three occasions for participants in both experimental groups. More specifically, they started with a general warm-up consisting of light cardiovascular exercise on a treadmill for approximately 10 min, followed by dynamic stretching of the upper and lower body for 10 min [32,33,34]. This was followed by a standardized warm-up set of the given exercise: i.e., 5 repetitions at ~50% 1-RM, followed by 1 set of 2–3 repetitions with loads at ~60–80% of estimated or known 1-RM. Gradually increasing weight was applied in order to perform 1 single full repetition until reaching 1-RM, with 3–5 min between trials. The five exercises were all performed in the same order on all three testing occasions: parallel back squat, barbell bench press, dumbbell military press, dead lifts, and barbell bent-over back, in this way providing adequate recovery time in moving from upper to lower body exercises. An 8-min passive recovery–resting period was kept for separating the 1-RM tests, and approximately 19 min separated upper and lower 1-RM exercises. Strength testing was performed using free weights, and was consistent with the guidelines established by the National Strength and Conditioning Association [28]. Two professional weight training specialists supervised all three testing sessions, and a 1-RM attempt was accepted when both coaches characterized it as valid.

### 2.5. Body Composition Assessment

To avoid any possible dietary confounding effects on body-composition assessment, all participants were instructed to record and follow their current dietary habits throughout the study. In each one of the three testing sessions, a 24 h food recall was taken by a registered Dietitian/Sports Nutritionist to check adherence (DAPA Measurement Toolkit, Cambridge, UK). Additionally, following the best possible practice for gathering body composition data via bioelectrical impendence (BIA) Body Composition Analyzer (MC-780MA Tanita, Tokyo, Japan), specific instructions were given to the participants. Specifically, prior to each measurement they were instructed not to consume alcohol or caffeine, and not to perform strenuous physical activity for at least 24 h, and to refrain from food and fluids intake for the three hours preceding the measurement. Measurements were taken at the same time of the day, with minimal clothing (shorts for men and shorts & crop tops for females). Female participants were prohibited from being measured during, or in proximity (±2 days) to, their menstruation or ovulation, due to possible water retention [35].

### 2.6. Statistical Analyses

Differences among the descriptive characteristics of the participants (age, height, weight, % body fat, fat mass and LBM) were tested with one-way between groups analysis of variance (1-way ANOVA). A mixed two-way 3 × 3 ANOVA (time [0, 6, 12 weeks] × group (LL, ML, CON)) was employed to compare the dependent variables between and within groups. When a significant main effect or interaction was observed (*p* < 0.05), a Tukey’s post-hoc test was performed. For pairwise comparisons, the magnitude of effect sizes was determined by calculating Hedge’s g (small effect = 0.20–0.49, medium effect = 0.50–0.79 and large effect ≥ 0.80). One-way ANOVA was performed to determine significant main effects on the absolute and percentage changes, compared with baseline, among the groups. The dependent variables were: (1) 1-RM strength in the five exercises used, (2) total fat mass (kg), (3) LBM, and (4) total bone mass throughout the intervention (week 0, week 6, and week 12 in the 3 experimental groups). The statistical analysis was carried out using the SPSS software (v.22.0, SPSS Inc., Chicago, IL, USA). Statistically significant differences were accepted at 0.05, and all results are reported as mean ± standard deviation.

## 3. Results

Volume load for all five exercises was similar in the LL and ML groups in the first 6 weeks of training (LL: 9481 ± 4400, ML: 9044 ± 4366 kg, main effect group: *p* = 0.851) and increased similarly, as it was adjusted according to the improvements in 1-RM of each exercise, in the last 6 weeks of training (LL: 10,202 ± 4810, ML: 9918 ± 4527 kg, main effect time: *p* = 0.001). No differences in volume load between the two conditions were identified for each exercise separately throughout the intervention. There were no baseline differences between groups in all demographics, body composition, and muscle strength variables (Table 1 and Table 2). Further, no significant change in any variable was observed in the CON group throughout the study.

### 3.1. Body Composition

**Figure 2:**
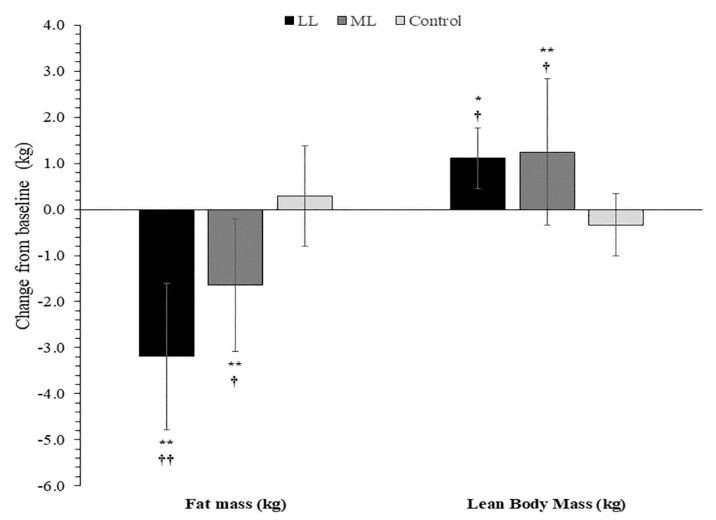
Changes in total fat mass (kg) and total lean body mass (kg), after 12 Weeks of HIFT for the low load group (LL), the moderate load group (ML) and the control group (CON), values are expressed as magnitudes of differences frοm baseline (±SD). Values expressed relative to the baseline values. The symbols * and **: *p* < 0.05 and *p* < 0.01 compared to corresponding baseline values; ^†^ and ^††^: *p* < 0.05 and *p* < 0.01 compared to CON.

No significant changes in body weight and bone mass were observed for any groups throughout the study (*p* = 0.72 and 0.86, respectively). In contrast, after 6 weeks of training, total body fat was similarly reduced in LL (−1.70 ± 1.09 kg, *p* = 0.0007) and ML (−1.38 ± 1.25 kg, *p* = 0.002) compared with their baseline values, as well as with the CON group values that remained unchanged (LL: *p* = 0.006, Hedge’s g = 1.73 and ML: *p* = 0.024, Hedge’s g = 1.33). At week 12, total body fat was further reduced for the LL group (*p* = 0.004), reaching −3.19 ± 1.59 kg (*p* < 0.001) compared to week 0 (% body fat at week 12 = 19.3 ± 6.6%). In contrast, no further decrease in total body fat was observed in ML from week 6 to week 12, with total fat loss from week 0 to week 12 reaching −1.64 ± 1.44 kg (*p* < 0.001, see Figure 2) (% body fat at week 12 = 18.6 ± 7.0%). These total body-fat reductions were greater (LL: *p* = 0.002, Hedge’s g = 1.09 and ML: *p* = 0.029, Hedge’s g = 1.41) than the non-significant change observed in the CON group (0.29 ± 1.08 kg, *p* = 0.998), and marginally greater in LL than in ML (*p* = 0.052, Hedge’s g = 0.99; Figure 2). 

Lean body mass increased only in the ML group (+1.05 ± 1.12 kg, *p* = 0.019) at the 6th week, while there was no change in the LL group (+0.45 ± 1.20 kg, *p* > 0.05) when compared with the respective baseline values. After 12 weeks of training, lean body mass increased similarly in the LL 0(+1.11 ± 0.65 kg, *p* = 0.03) and ML (+1.25 ± 1.59 kg, *p* = 0.002) groups respectively, compared with both their baseline values. These changes were greater (LL: *p* = 0.04 for LL, Hedge’s g = 2.10 and ML: *p* = 0.02, Hedge’s g = 1.14) compared with the non-significant change observed in the CON group (−0.34 ± 0.67 kg, *p* = 0.992; Figure 2).

### 3.2. 1-RM Strength in Different Resistance Exercises

The two-way mixed ANOVA showed a statistically significant increase in muscle strength in the back squat exercise at week 6 only for the ML group (ML: + 5.62 ± 4.41%, *p* < 0.001) compared with baseline values, but not compared with the CON group (*p* > 0.05). Both groups presented statistically significant increases in 1-RM at week 12 (LL: 10.08 ± 4.55%, *p* < 0.001; ML:12.57 ± 4.76%, *p* < 0.001) compared with baseline. However, only the ML group reached a statistical significance compared with the CON group (*p* = 0.05, Hedge’s g =1.15).

In the bench press 1-RM, the analysis revealed that after 6 weeks of training, only the LL group increased 1-RM strength (LL: 8.83 ± 7.40%, *p* < 0.001) compared with baseline but not with the CON group (*p* > 0.05). At week 12, both groups demonstrated significant improvements in bench press 1-RM (LL: 11.78 ± 6.88%, *p* < 0.001; ML:15.99 ± 6.06%, *p* < 0.001) compared with baseline and with the CON group (LL: *p* = 0.03, Hedge’s g = 1.18; ML: *p* = 0.01, Hedge’s g = 1.47). 

Regarding the bent-over row 1-RM performance, the analysis showed a statistically significant increase in muscle strength at week 6 only for the ML group (ML: 5.68 ± 7.51%, *p* = 0.02) compared with baseline values, but not with the CON group (*p* > 0.05). Nevertheless, both the ML and LL groups displayed statistically significant increases in 1-RM at week 12 (LL: 8.89 ± 5.89%, *p* < 0.001; ML: 9.30 ± 4.81%, *p* < 0.001), compared with baseline values and the CON group (LL: *p* = 0.002, Hedge’s g = 1.48; ML: *p* = 0.001, Hedge’s g = 1.95). 

Both groups demonstrated significant increases in dead lift 1-RM at both week 6 (LL: 5.67 ± 3.47%, *p* = 0.01; ML: 8.53 ± 3.36%, *p* < 0.001) and week 12 (LL: 10.03 ± 5.18%, *p* < 0.001; ML: 10.84 ± 4.96%, *p* < 0.001) compared with their corresponding baseline values, as well as with the CON group at both week 6 (LL: *p* = 0.01, Hedge’s g = 1.47; ML: *p* = 0.002, Hedge’s g = 1.99) and week 12 (LL: *p* = 0.04, Hedge’s g = 1.31; ML: *p* = 0.02, Hedge’s g = 1.31). 

There was no significant group x time interaction (*p* = 0.072) or group main effect (*p* = 0.281) for the military dumbbell shoulder press 1-RM. However, there was a time effect (*p* < 0.001), indicating that all groups improved by 3.25 ± 6.70 to 12.01 ± 9.40%.

## 4. Discussion

The purpose of the present study was to examine the effects of HIFT training with different resistance loads (i.e., LL: 30% 1-RM vs. ML: 70% 1-RM) on body composition and maximal strength performance. The main finding of the present study was that both loads decreased fat mass and increased lean body mass (LBM) after 12 weeks of HIFT training (Figure 2). Interestingly, the LL group showed a marginally greater loss of fat mass compared to the ML group after 12 weeks of training (*p* = 0.052, Hedge’s g = 0.99). In addition, HIFT using either load equally increased 1-RM strength in four out of five resistance exercises (back squat, dead lift, bent-over row, and bench press).

Even though it was expected that both groups would show improvements, it was hypothesized that the ML group would have greater improvements compared with the LL group in LBM and maximal strength. To our knowledge, only a few studies have previously examined the effect of HIFT training on body composition and maximal strength performance for a relatively long period of training (8–16 Weeks) [16,19,20,21,36,37]. In the present study we demonstrated that three HIFT sessions per week for 12 weeks (a total of 36 sessions), led to improvements in performance and overall body composition by reducing body fat mass and increasing LBM. 

The observed changes in fat mass are consistent with the majority of the existing literature implementing a similar protocol. In a recent study, Heinrich et al. [15] found that cancer survivors reduced fat mass, and they also observed an increase in LBM after HIFT training. In that study, which lasted only five weeks, participants demonstrated a reduction of −15% in total body fat mass. Similarly, Fealy et al. [38] showed a −5.5% reduction in fat mass after 6 weeks of HIFT training (three sessions per week) in thirteen obese individuals having been diagnosed with type 2 diabetes. In accordance with these findings, on the sixth week of the present study we observed a larger reduction in total fat mass (by 11%) for both the LL and ML group respectively, which was significantly different compared with the CON group. Interestingly, in the second half of the intervention (Week 6 to Week 12), both groups continued showing a significant loss of body fat mass compared to baseline values, with the LL group showing marginally greater changes compared to the ML in the second half of the intervention (*p* = 0.052). These findings are in accordance with longer studies examining the effect of HIFT on body fat mass [16,20]. One possible explanation for the tendency of the LL group to lose more fat at the end of the intervention may be the number of repetitions performed. While both groups performed the same circuit program with equal volume load (repetitions x load), the LL group performed 24–28 repetitions per set, which is a characteristic of muscle-endurance protocols. Similar protocols have shown that lower loads of resistance training coupled with a high number of repetitions have demonstrated significant body fat losses [39,40]. 

The observed changes in body fat mass, without significant changes in total body mass, reflect the increase in LBM. Both groups demonstrated statistically significant enhancements in LBM at the end of the HIFT training by +1.11 ± 0.65 (2.1 percent) and 1.25 ± 1.59 (2.2 percent) for the LL and ML groups respectively, both significantly different compared with the CON group. These findings are consistent with previous reports examining HIFT on similar-level trained individuals, such as recreationally active individuals by ~3.3% [37] or trained individuals by ~2.0% [19]. In contrast, Feito et al. [17] demonstrated significant reductions in % of fat mass, but not in bone-free lean mass, after 16 weeks of CrossFit training, in relatively experienced individuals. Notably, these researchers mentioned that since the individuals had been exposed to a similar type of training for an average of 16 months, it was possible that they were under a “maintenance’’ stage, with no intention to further modify their body composition. However, in the present study, the observed increase in LBM could be attributed to the fact that the participants were recreationally trained and had not taken part in heavy resistance training prior to the study.

Another interesting finding in the present study is that after six weeks of HIFT, only the ML group demonstrated a significant increase in LBM by +1.05 ± 1.12 kg or ≈2% (*p* = 0.0186) compared with baseline values. Muscle hypertrophy is usually minimal during the first 4 weeks of a RT program, and the increases in muscle strength during this period are mostly due to neural adaptations [8]. Although recent studies have showed that RT-induced hypertrophy can occur from the very early stages of a training program [41] the accretion of muscular protein becomes evident only after six weeks or more [42]. Research has shown that strength training when the intensity of exercise is between 70–80% of 1-RM leads to superior effects in intramuscular coordination and higher specific hypertrophy compared with lower loads (<40% 1-RM) [43]. In addition, recently Damas et al. [44] proposed that hypertrophy following 3–4 weeks of training is not indicative of the magnitude of a ‘“true” hypertrophy, and could, in part, show edema-induced muscle swelling from unaccustomed exercise. Unfortunately, bioelectrical impedance (BIA), a method that we used in the present study, does not differentiate LBM, muscle edema or muscle glycogen. Nevertheless, even when muscle hypertrophy is directly measured, increases in muscle fiber cross-sectional area are significant from pre-training only after 7 weeks of training [45]. It could therefore be hypothesized that due to the “heavier” strain on the muscle in the ML group, hypertrophy was faster compared with the LL and therefore demonstrated superior results in the first half of the study. 

The faster muscle-protein accretion in ML might also be explained by a fiber-type-dependent effect of resistance training against different loads. Schoenfeld et al. [30] mentioned that type II muscle fiber has been shown to have approximately 50% greater capacity for hypertrophy compared to type I. Research in this area has been relatively biased, with the majority of studies examining RT intensities >60% 1-RM. Therefore, we could hypothesize that due to the fatigue-resistance characteristic of type I fibers, more time under tension associated with low-load training is needed to fully activate these fibers. This has been supported by Netreba et al. (2007) who found that training with loads equal to 80–85% 1-RM favors increases in cross-sectional area of fast-twitch fibers, whereas training at lower intensities (50% 1-RM) produces greater enhancements in the slow-twitch fiber type I cross-sectional area [46]. Furthermore, the use of light loads has been found to present a greater muscle-endurance-type load, greater total integrated muscle electromyographic activity, and more time under tension than higher loads when the participants perform the sets to momentary failure [29,47]. This compensates the lower muscle activity during low load training, activating also some type II fibers towards the end of the set [48]. This compensation is the main reason why lower loads may provide a similar hypertrophy, if they are performed to failure, compared with the heavier loads. Therefore, it is possible that in the present study, the observed similar increase in lean body mass between the two equaled protocols after 12 weeks of HIFT training may have been delivered by hypertrophic responses in different type fibers, a hypothesis that requires further investigation. 

Strength performance gains were similar between groups, with four out of five 1-RM strength tests showing statistically significant increases compared with baseline and with the CON group. These results are in accordance with previous studies proposing HIFT as an effective protocol to improve maximal muscle strength [20,49]. In contrast to our hypothesis, no statistically greater increases in maximal strength were identified in the ML group. Although higher-load training is generally accepted as producing superior results in maximal strength, we did not observe any differences between the two intervention groups (ML vs. LL), although the ML group provided non-significant but superior results compared with the LL group in the bench press 1-RM test (16.0 ± 6.1% vs. 11.8 ± 6.9%), the bent-over row 1-RM test (9.3 ± 4.8% vs. 8.9 ± 5.9%) and the dead lift 1-RM test (10.8 ± 5.0% vs. 10.0 ± 5.2%). The results of the present study are in accordance with the results of Dinyer et al. [50], who found similar increases in 1-RM strength, regardless of training load (30% 1-RM vs. 80% 1-RM) for untrained women. However, a difference between the present study and the study of Dinyer et al. [50] is that in the previous study, participants performed the sets till the instant of exhaustion, whereas in the present study, they performed as many repetitions as they could within 30 s, and not to failure. However, a recent study that compared the effects of resistance training until failure and non-failure on LBM, maximal strength, and muscle activation in trained individuals, found similar increases in hypertrophy and 1-RM in leg press and leg extension exercises [51]. Thus, it remains to be examined whether the similarities in strength gains are due to the fitness level of the participants, who were not highly trained, and/or the duration of the study, with possible differences emerging after 12 weeks of training. Maximal strength does not only depend on muscle mass, which was equally increased in both groups as suggested by changes in LBM, but may also be enhanced due to neural adaptations [26,27]. Nevertheless, it is yet unknown as to how the use of different loads during HIFT might influence neural parameters, such as the rate of motor unit firing, the frequency of doublet firing, synchronization of motor units, and coactivation ratios of agonist–antagonist muscles [30].

## 5. Conclusions

In conclusion, this study showed that twelve weeks of HIFT with either low or moderate loads resulted in an equal increase in LBM and 1-RM strength. However, fat loss tended to be greater after 12 weeks in the LL compared with ML. These results could help strength and conditioning coaches to design HIFT training programs depending on the period and the goal of training session. For example, HIFT with low loads may be recommended when an athlete is returning from an injury and the use of moderate loads is prohibited. Furthermore, HIFT training with low loads could be used in the beginning until the middle of the preparatory period of sports training, or in the general population, when the goal is to improve body composition and maximal strength without imposing high forces on the musculoskeletal system. Due to their similar effects on body composition and strength, HIFT with LL and ML may be alternated during a training period, in order to periodize the stimulus and avoid training monotony, at least in the general population.

## Figures and Tables

**Table 1:** Descriptive characteristics and body composition of the participants at baseline (mean ± SD).

	LL	ML	CON	*p* Value
Age (years)	31.4 ± 7.1	27.5 ± 5.1	30.3 ± 7.5	0.333
Weight (kg)	71.1 ± 12.4	70.0 ± 14.7	74.7 ± 14.7	0.788
Height (m)	1.72 ± 0.1	1.68 ± 0.1	1.71 ± 0.1	0.890
BMI (kg/m^2^)	24.3 ± 1.4	24.2 ± 3.2	25.6 ± 2.8	0.965
Body Fat (%)	23.3 ± 6.8	21.1 ± 7.0	27.5 ± 4.1	0.624
Fat Mass (kg)	16.3 ± 4.4	14.8 ± 6.5	20.4 ± 4.0	0.528
Lean Body Mass (g)	52.0 ± 11.4	52.4 ± 11.5	51.1 ± 11.4	0.972
Bone Mass (kg)	2.76 ± 0.5	2.77 ± 0.5	2.76 ± 0.5	0.763

LL: low-load resistance training 30% 1-RM; ML: moderate-load resistance training 70% 1-RM; CON: control group, values are mean (±SD).

**Table 2:** Maximal strength (1-RM) of the participants in the three groups at baseline and after 12 weeks of training (mean ± SD).

	LL		ML		CON	
	Baseline	Week 12	Baseline	Week 12	Baseline	Week 12
Bench Press (kg)	51.0 ± 26.1	58.0 ± 30.6 **^†^	47.3 ± 25.3	55.0 ± 28.3 **^†^	44.4 ± 12.7	45.0 ± 14.5
Back Squat (kg)	77.5 ± 33.9	85.5 ± 36.3 **^†^	71.5 ± 34.2	80.8 ± 38.8 **^†^	56.8 ± 12.9	59.8 ± 15.3
Dead Lift (kg)	86.0 ± 36.3	95.5 ± 40.7 **^†^	79.2 ± 36.4	88.1 ± 40.2 **^†^	68.1 ± 19.4	70.1 ± 20.6
Bent-Over Row (kg)	50.5 ± 21.9	55.0 ± 23.1 **^††^	53.8 ± 24.5	58.5 ± 24.9 **^††^	45.6 ± 14.0	45.6 ± 15.3
Shoulder Press (kg)	36.5 ± 15.1	41.5 ± 19.0	36.5 ± 15.2	41.2 ± 19.1	27.8 ± 9.2	28.8 ± 9.8

LL: low-load resistance training 30% 1-RM; ML: moderate-load resistance training 70% 1-RM; CON: control group, values are mean (±SD and are expressed relative to the baseline values, **: *p* < 0.01 compared to corresponding baseline values; ^†^ and ^††^: *p* < 0.05 and *p* < 0.01 compared to the corresponding value of CON.

## Data Availability

Data available on request.
